# Understanding Data Use in Community Coalitions: Insights from a CFIR 2.0-Informed Study of the Ohio HEALing Communities Initiative

**DOI:** 10.1007/s10597-026-01617-6

**Published:** 2026-04-28

**Authors:** Kathryn Lancaster, Cheyenne Wagi, John Myers, Maria Pacheco Garrillo, Sylvia Ann Ellison, Ramona Olvera, Bridget Freisthler

**Affiliations:** 1 Department of Implementation Science, Wake Forest University School of Medicine, Winston-Salem, USA; 2 Center for Biostatistics, College of Medicine, The Ohio State University Wexner Medical Center, Columbus, USA; 3 College of Public Health, The Ohio State University, Columbus, USA; 4 The Ohio State University Wexner Medical Center, Columbus, USA; 5 BMEA Division of Bioethics, College of Medicine, The Ohio State University Wexner Medical Center, Columbus, USA; 6 College of Social Work, University of Tennessee at Knoxville, Knoxville, USA

**Keywords:** Community coalitions, CFIR 2.0, Mixed-methods, Data-informed decision making, Opioid use disorder, Data coordination, Evidence-based practices

## Abstract

Community coalitions play a central role in implementing evidence-based strategies to reduce opioid overdose deaths. However, their ability to effectively use community-level data in decision-making varies widely and is shaped by multiple contextual factors. This study examined how coalitions participating in the Ohio HEALing Communities Study engaged with data to support implementation of opioid-related interventions, including overdose education and naloxone distribution, and medications for opioid use disorder. We conducted a mixed-methods study guided by the Consolidated Framework for Implementation Research (CFIR 2.0) to explore data use within 18 community coalitions in Ohio. Quantitative data were collected from coalition members via REDCap surveys administered at four timepoints (2019–2023), assessing knowledge, confidence, and perceptions related to data use. Qualitative data were obtained through in-depth interviews with coalition members and data coordinator reflections. Descriptive statistics summarized survey responses, and thematic analysis was used to identify patterns in qualitative data. Findings were derived through an integrated mixed-methods approach, combining survey trends with in-depth interview themes and contextual reflection data. Coalition members reported increased knowledge and confidence in tracking opioid-related interventions over time. By 2023, over 90% of respondents supported learning about naloxone and medications for opioid use disorder and felt confident in their coalition’s ability to monitor progress. However, the extent to which data informed decision-making varied. Findings offer practical insights to enhance coalition capacity for sustained, equitable, and data-informed implementation in complex public health settings. Data coordinators played a key role in interpreting and applying data. Barriers included limited data literacy, inconsistent meeting structures, fragmented external systems, and policy misalignment. While some coalitions used data to drive equitable resource allocation and advocacy, others relied on it primarily for reporting and funding purposes. Findings highlight the importance of aligning data strategies with coalition capacity, infrastructure, and culture. Sustained, meaningful use of data requires not only technical tools but also investment in coalition readiness, leadership engagement, and cross-sector partnerships. These results provide actionable insights for supporting community-driven, data-informed implementation of opioid prevention strategies in real-world settings.

## Introduction

Ohio has been among the states hardest hit by the opioid epidemic, with the second highest opioid overdose death rate in the nation in 2017 and consistently ranking among the top ten states through 2023 (Centers for Disease Control and Prevention, 2025a, 2025b; KFF, 2023). This crisis has strained healthcare systems, destabilized communities (Darolia & Heflin, 2022), and underscored the urgent need for coordinated, data-driven approaches to prevention, treatment, and recovery support (Chandler et al. 2020a). The HEALing Communities Study (HCS) sought to address this crisis by advancing the evidence base for community engagement as a strategy to support the implementation and sustainability of evidence-based practices (EBPs) in areas most affected by opioid-related harm (Freedman et al. 2025; Sprague Martinez et al. 2020a).

The HCS employed the Communities That HEAL intervention, which integrates three key components: (1) coalition-led community engagement to build capacity and foster collaboration (Sprague Martinez et al. 2020a); (2) the Opioid-overdose Reduction Continuum of Care Approach, which offers a structured menu of EBPs to reduce opioid use disorder (OUD) and overdose deaths (Winhusen et al. 2020a); and (3) targeted communication campaigns to reduce stigma and increase awareness and uptake of EBPs (Lefebvre et al., 2020). This integrated approach highlights the potential for data-informed, coalition-driven strategies to promote sustainable systems change.

Community-based research proposes that community-level data plays a critical role in tailoring interventions to local needs (Community Tool Box, 2025; Levin & Schneir, 2015). For HCS Ohio, community data provided insights into opioid use patterns, overdose rates, treatment access, and disparities (Slavova et al., 2020). Coalitions use these data to inform strategy, monitor progress, and identify gaps in care (Wu et al., 2020). In practice, coalitions use these data to identify areas with elevated overdose burden (e.g., by ZIP code) and gaps along the ORCCA continuum, such as mismatches between OUD prevalence and local MOUD availability or limited OEND reach in high-burden communities (Young et al., 2022; Zang et al., 2021). These insights help coalitions prioritize target settings and subregions, set locally relevant implementation goals, and track changes over time to determine where coverage is lagging and where additional coordination or resources are needed. The value of data lies in its relevance and trustworthiness; its utility, in its capacity to drive action and improve outcomes.

Community-level data must be made accessible and actionable; therefore, the development and implementation of community-facing data dashboards (El-Bassel et al., 2021; Stahlman et al., 2025) and models like Community-Engaged Data Science have further demonstrated the importance of embedding data engagement roles, such as data coordinators who help to facilitate the translation of data into local action. Recent evaluations underscore that dashboards alone are insufficient without tailored, iterative, and trusted facilitation roles and infrastructure (Fareed et al., 2024; Olvera et al., 2024). The Community-Engaged Data Science model, employed in Ohio as part of HCS, offers a stepwise, participatory process that leverages human-centered design and community-based participatory research to align data tools with community priorities and workflows (Olvera et al., 2024).

Coalitions serve as the operational backbone of HCS, uniting stakeholders such as public health officials, healthcare providers, policymakers, and community members to implement Communities That HEAL (Sprague Martinez et al. 2020b). However, the effectiveness of coalition-led decision-making is shaped by multiple contextual factors, including external partnerships, IT infrastructure, organizational learning culture, and individual data literacy (Olvera et al., 2024). To assess how these factors influence coalition capacity, this study applies the Consolidated Framework for Implementation Research (CFIR) 2.0, which provides a structured lens for examining outer and inner setting characteristics, intervention design, implementation processes, and individual-level factors that shape coalition engagement with community-level data (Damschroder et al., 2022). CFIR 2.0 organizes implementation determinants into five domains, innovation characteristics, outer setting, inner setting, individuals, and implementation process, allowing researchers to systematically assess how multilevel contextual factors influence adoption, integration, and sustainment. In this study, CFIR 2.0 guided the analysis, helping us examine how coalition structure, leadership, infrastructure, and member capability shaped engagement with community-level data over time.

Using a CFIR 2.0-guided mixed-methods approach, we explore how coalitions navigate the challenges and opportunities of data-driven decision-making within HCS. Specifically, we assess (1) the value and utility of community-level data in guiding coalition EBPs for increasing overdose education and naloxone distribution (OEND) and medications for opioid use disorder (MOUD); (2) coalition perspectives on data adoption over time, identifying key outer and inner setting factors influencing usability and integration; and (3) variations in coalition approaches across diverse settings, highlighting challenges and best practices for sustaining data-informed strategies. By applying CFIR 2.0, we provide insights into how coalitions evolve in their data-driven decision-making capacity, the role of external partnerships in sustaining data access, and the challenges of aligning data with real-world intervention needs. These findings will inform efforts to strengthen coalition-led, data-informed public health strategies for opioid overdose prevention.

## Methods

### Trial Design

The HEALing Communities Study (HCS) was a multisite, wait-listed, parallel-group cluster randomized trial conducted across 67 communities in four states (KY, MA, NY, and OH) heavily impacted by the opioid epidemic (Chandler et al. 2020b). Designed to evaluate the effectiveness of a community-level intervention to reduce opioid overdose deaths, HCS required participating communities to form or sustain local coalitions that prioritized EBPs, including OEND, MOUD, and safer opioid prescribing. The Communities That HEAL intervention emphasized community engagement through HCS coalitions as a key implementation strategy, involving stakeholders across healthcare, behavioral health, criminal legal systems, and community organizations. The trial began for Wave 1 communities in January 2020 and concluded in June 2022. The wait-listed communities received the Communities That HEAL intervention in Wave 2 from July 2022 through December 2023. In Ohio, 18 counties and their respective HCS community coalitions from 19 randomized counties, participated, including rural and urban sites with high overdose mortality rates.

HCS Ohio hired and trained a field team of community focused staff, often residents from the communities with prior community engagement or coalition experiences, to work closely with coalitions to facilitate intervention implementation, communication activities, and data coordination. Some field team staff were hired from embedded coalition members whereas others were new to the opioid coalitions. Individual HCS field teams were assigned to each participating coalition and worked closely with community-selected coalition leads and dedicated champions. Field teams supported pre-existing coalition structures and personnel when aligned with intervention requirements and financially incentivized study champions when allowable. A key field team member was the data coordinator whose role was to engage coalitions in use of study-supplied and coalition-collected data for decision-making and EBPs tracking and evaluation. Study-supplied data was accessible through a password-protected portal that included a data dashboard with each community. Details on the data available and the community engagement with the dashboards can be found elsewhere (Fareed et al., 2024; Wu et al., 2020).

## Study Design

This mixed-methods study integrated quantitative and qualitative data to understand how coalitions used data to support EBPs’ selection, implementation, and sustainment. Guided by CFIR 2.0, the design intended to capture both community-level patterns and the contextual factors shaping data engagement and use across time.

## Data Collection and Analytic Approach

We employed a qualitatively driven, parallel design to examine coalition engagement with data systems during implementation of the HCS in Ohio. Quantitative and qualitative data were collected concurrently at four timepoints: November–December 2019, May–June 2021, May–June 2022, and November–December 2023 (Table 1) (Knudsen et al., 2020).

**Table 1:** Data collection and analysis overview

**Measure**	**T0**	**T1**	**T2**	**T3**	**T4**	**Data Type**	**Analysis Approach**	**Framework(s)**	**Data Source Size**
Coalition Member Surveys	X	X	X		X	Quantitative	Descriptive statistics	CFIR 2.0 domains	N = 197 wave 1; 195 wave 2
Coalition Member Interviews			X		X	Qualitative	Thematic analysis	Inductive + CFIR 2.0	N = 92 interviews
Data Coordinator Reflections				✓		Qualitative	Content synthesis	CFIR (Outer/Inner)	DC: N=7; Counties represented: N=11

T0 = Nov–Dec 2019 (baseline); T1 = May–Jun 2021 (Wave 1 midpoint, Wave 2 baseline); T2 = May–Jun 2022 (Wave 1 endpoint); T3 = Nov–Dec 2023 (Wave 2 endpoint); T4 = post-intervention follow-up for Wave 1

Quantitative data were collected via REDCap surveys sent by email invitations to all active coalition members, as identified by leadership and attendance records. Details of the survey data collection are reported elsewhere (Knudsen et al., 2020). To increase responses, researchers sent reminder emails and made announcements at coalition meetings. The approximately 45-minute survey assessed coalition knowledge of OEND and MOUD, perceived community need, confidence in tracking implementation progress, and attitudes toward data use in decision-making. Most items were measured using Likert-type response scales (e.g., strongly disagree to strongly agree). Because coalition membership changed over time, surveys captured repeated cross-sectional snapshots rather than individual longitudinal trajectories. For this analysis, we focused on items related to knowledge, confidence, and data engagement, and summarized responses using descriptive statistics (percentages and proportions) to examine trends across the four time points.

Qualitative data were gathered through individual in-depth interviews with a sample of coalition members across the same four timepoints. Details on the sampling, interviews, and procedures for the initial deductive coding are reported elsewhere (Drainoni et al., 2022; Knudsen et al., 2020; McAlearney et al., 2023). In brief, a purposive sample of coalition members was selected to ensure representation across behavioral health, healthcare, and criminal justice sectors. Coalition leads and data champions were included based on availability. For each interview, a trained interviewer explored coalition processes, decision-making, data engagement, and sustainability using a semi-structured guide. The interviews analyzed in this study were scheduled for one hour, conducted over Zoom, and were transcribed verbatim.

Additionally, case-level reflections and documentation were contributed by HCS-funded data coordinators, who were locally embedded staff supporting coalition data engagement. These reflections included structured reports, meeting summaries, and field notes, offering insight into coalition-level activities such as dashboard use, data sharing, and advocacy.

## Analytic Procedures

Survey data were summarized using descriptive statistics to assess trends in coalition knowledge, confidence, and support for OEND and MOUD over time. Results were reviewed by wave to assess differences in coalition readiness and data engagement.

For the qualitative data analysis, a postdoctoral researcher and a PhD student conducted inductive coding and reflective thematic analysis (Clarke & Braun, 2018) of data from interview transcripts at two timepoints, aligning with the conclusion of the Communities That HEAL intervention for Wave 1 and Wave 2 (timepoint 3 and timepoint 4, respectively). Initial codes were derived directly from the data, with both analysts independently coding two interviews to create a working codebook. After establishing consensus on the codebook, the researchers met weekly to iteratively discuss any necessary refinement of the codebook and to consolidate initial themes. The two researchers developed final themes through consensus and with input from all co-authors to ensure analytic rigor and depth. NVivo 12 software supported qualitative data management and analysis. To explore potential differences in perspectives, we organized quotes by wave, sector, and urbanicity.

Following inductive coding and theme development, we conducted a secondary, theory-informed mapping process to align emergent themes with CFIR 2.0 domains and constructs. An analyst independently reviewed the finalized themes and assigned them to relevant CFIR domains based on construct definitions provided in the CFIR 2.0 framework. The research team then met to compare classifications, discuss areas of overlap or ambiguity, and reach consensus through iterative discussion. In cases where themes spanned multiple domains, we prioritized the domain most central to the mechanism described while noting cross-domain influences in interpretation. This process enhanced conceptual clarity and ensured consistent application of CFIR 2.0 across findings.

Data coordinator reflections were analyzed using content synthesis to identify implementation challenges and adaptations at the coalition level. Although quantitative and qualitative data were not formally integrated during analysis, survey trends were used to contextualize qualitative insights. This design aligns with established principles of qualitatively driven parallel designs, in which qualitative inquiry anchors interpretation while quantitative data augment explanatory depth (Creswell & Plano Clark, 2017). In reporting results, we explicitly indicate whether findings are derived primarily from survey data, interview themes, or data coordinator reflections.

We analyzed survey responses from Wave 1 (*n* = 197) and Wave 2 (*n* = 195) coalition members. The qualitative sample included 92 interviews, evenly divided between Wave 1 (*n* = 46) and Wave 2 (*n* = 46), with nearly half from rural coalitions (45.7%; *n* = 42). Of the interviewees, 28 were identified as champions, community coalition members with specific interest in data. Sector representation included behavioral health (*n* = 34), healthcare (*n* = 34), criminal legal (*n* = 13), and other (*n* = 11). Most qualitative participants were white (90.2%; *n* = 83), non-Hispanic (97.8%, *n* = 90), females (58.7%, *n* = 54), with an associate’s degree or higher education (90.2%, *n* = 83). Data coordinator reflections included 7 data coordinators representing 11 counties.

## Results

### Innovation: Design and Complexity of Data-Driven Strategies

Coalitions varied in their ability to implement data-informed decision-making, reflecting differences in the complexity of data use and the design quality of support available (*Complexity; Design Quality and Packaging*). All communities were expected to develop a Data Action Plan to guide local data-sharing infrastructure and strategy. Interview data and data coordinator reflections suggested that coalitions that embraced and effectively leveraged this structured tool were better equipped to integrate data into decision-making. In contrast, others struggled with the ambiguity or fragmentation of available tools, indicating a need for more accessible and context-appropriate data tools.

Knowledge of OEND and MOUD improved over time, reflecting growing exposure to data resources (Table 2). In Wave 1, the percentage of respondents reporting their coalition was knowledgeable about OEND rose from 70.3% in 2019–2020 to 93.2% in 2023, while MOUD knowledge rose from 55.8% to 89.2%. In Wave 2, similar trends were seen, with MOUD knowledge rising from 54.3% to 73.1%. Despite this progress, how coalitions integrated data into decision-making remained variable.

**Table 2:** Coalition knowledge and support for OEND and MOUD across timepoints and waves

**Question**	**Wave**	**Survey**	**Not at all**	**Slight / Moderate Extent**	**Great/ Very great extent**	**Refused**
This coalition is knowledgeable about OEND in this community.	1	Baseline	2 (1.7%)	31 (26.3%)	83 (70.3%)	2 (1.7%)
		Follow-up	0 (0%)	3 (6.8%)	41 (93.2%)	0 (0%)
	2	Baseline	2 (1.8%)	28 (24.6%)	84 (73.7%)	0 (0%)
		Follow-up	0 (0%)	6 (8.7%)	62 (89.9%)	1 (1.4%)
This coalition is knowledgeable about MOUD/MAT in this community.	1	Baseline	5 (4.8%)	36 (34.6%)	58 (55.8%)	5 (4.8%)
		Follow-up	0 (0%)	3 (8.1%)	33 (89.2%)	1 (2.7%)
	2	Baseline	6 (5.7%)	40 (38.1%)	57 (54.3%)	2 (1.9%)
		Follow-up	0 (0%)	14 (20.9%)	49 (73.1%)	4 (6%)
This coalition supports efforts to learn more about OEND in this community.	1	Baseline	1 (0.8%)	19 (16.1%)	96 (81.4%)	2 (1.7%)
		Follow-up	0 (0%)	5 (11.4%)	39 (88.6%)	0 (0%)
	2	Baseline	0 (0%)	29 (25.4%)	85 (74.6%)	0 (0%)
		Follow-up	1 (1.4%)	4 (5.8%)	64 (92.8%)	0 (0%)
This coalition supports efforts to learn more about MOUD/MAT in this community.	1	Baseline	6 (5.8%)	25 (24.3%)	69 (67%)	3 (2.9%)
		Follow-up	0 (0%)	1 (2.7%)	35 (94.6%)	1 (2.7%)
	2	Baseline	7 (6.7%)	36 (34.3%)	57 (54.3%)	5 (4.8%)
		Follow-up	0 (0%)	14 (20.9%)	50 (74.6%)	3 (4.5%)

Some coalitions used data strategically, applying it to real-world resource allocation, as one participant noted,

*“The data was also able to allow the consortium to look at areas in the county that had higher rates of overdoses per capita. So that they could kind of target that area*,* you know through ZIP codes.”* (Wave 1, healthcare, rural).

The Data Action Plan helped some coalitions operationalize such efforts by providing a shared framework for decision-making:

“*And the Data Action Plan that we developed through…the data committee with the HEALing Communities*,* we are always*,* you know*,* anytime you make decisions*,* it was open and there we were looking at it to kind of comb it to help drive us in the best direction*.” (Wave 1, healthcare, rural).

From the data coordinator reflections, a Wave 2 rural county data coordinator described how dashboard data motivated local advocacy for MOUD access. A coalition member was particularly struck by Medicaid data on the HCS county dashboard showing that 100 residents diagnosed with opioid use disorder were receiving MOUD, despite having no MOUD providers in the county. Recognizing that these individuals were traveling outside the county for treatment, the coalition member consistently used the data to highlight a clear gap in local services. This advocacy ultimately contributed to the Mental Health and Recovery Services Board issuing a request for proposals to bring a MOUD provider into the county.

However, some coalition members found the available data overwhelming or disconnected from community decision-making processes, as this participant explained:

“*We’re data rich too and it’s one of the first things we did when we started the coalition*,* but…I don’t think we use data… I don’t necessarily know if HCS did anything to really change how we do data and it’s not just us. It’s across the country. Data is not being used appropriately*;” (Wave 1, criminal legal, urban).

A Wave 1 urban county data coordinator observed that in the community in which they worked, the structure and planning of coalition meetings influenced how data were used. Data were not consistently incorporated into coalition discussions, despite early suggestions from some members that data should inform all coalition topics and efforts. However, meeting agendas were often developed at the last minute, which limited opportunities to make data a regular part of coalition activities.

## Outer Setting: Community Data Needs, Partnerships, and Policy Influences

Outer setting factors, including community needs, coalition diversity, and policy influences, shaped coalitions’ ability to access, interpret, and use data effectively. While most coalitions faced high community demand for OEND, interview narratives and data coordinator reflections indicated that coalitions with strong cross-sector relationships and aligned goals were more likely to act on data. Others encountered barriers such as fragmented systems or policy misalignment that limited data use in strategic planning.

Coalitions recognized the need for increased data availability and public education regarding opioid-related interventions (Table 3). By 2023, over 94% of respondents across both waves agreed or strongly agreed that their communities needed more information about OEND and MOUD effectiveness. One participant emphasized both the urgency of public access to data and the challenges of accessing usable available data, stating,

**Table 3:** Coalition member perceptions of data needs and confidence in implementing opioid-related interventions across waves and timepoints

**Question**	**Wave**	**Survey**	**Strongly disagree / Disagree**	**Neutral**	**Agree / Strongly agree**	**Refused**
Thinking about the opioid epidemic in this community and the current resources available to address it, to what extent do you agree or disagree that your community needs… More information for the general public about the effectiveness of OEND.	1	Baseline	1 (0.7%)	7 (5.2%)	127 (94.1%)	0 (0%)
		Follow-up	0 (0%)	3 (6.8%)	41 (93.2%)	0 (0%)
	2	Baseline	3 (2.3%)	6 (4.7%)	117 (91.4%)	2 (1.6%)
		Follow-up	2 (2.9%)	2 (2.9%)	65 (94.2%)	0 (0%)
Thinking about the opioid epidemic in this community and the current resources available to address it, to what extent do you agree or disagree that your community needs… More information for the general public about the effectiveness of MOUD/MAT	1	Baseline	3 (2.5%)	5 (4.1%)	112 (91.8%)	2 (1.6%)
		Follow-up	0 (0%)	0 (0%)	36 (97.3%)	1 (2.7%)
	2	Baseline	3 (2.7%)	8 (7.1%)	101 (89.4%)	1 (0.9%)
		Follow-up	0 (0%)	2 (2.9%)	65 (95.6%)	1 (1.5%)
People in this coalition feel confident that they can keep track of progress in implementing OEND.	1	Baseline	2 (1.7%)	16 (13.3%)	100 (83.3%)	2 (1.7%)
		Follow-up	3 (6.7%)	6 (13.3%)	36 (80.0%)	0 (0%)
	2	Baseline	7 (5.8%)	20 (16.5%)	93 (76.9%)	1 (0.8%)
		Follow-up	0 (0%)	4 (5.8%)	63 (91.3%)	2 (2.9%)
People in this coalition feel confident that they can keep track of progress in implementing MOUD/MAT.	1	Baseline	13 (12.4%)	26 (24.8%)	64 (61%)	2 (1.9%)
		Follow-up	5 (12.8%)	4 (10.3%)	29 (74.4%)	1 (2.6%)
	2	Baseline	19 (17.8%)	27 (25.2%)	60 (56.1%)	1 (0.9%)
		Follow-up	1 (1.5%)	7 (10.4%)	54 (80.6%)	5 (7.5%)

*“I can’t wait to dig into all your data and all your publications and everything. Hopefully that will be available*,* you know*,* in the public realm for purposes. We desperately need the data on this stuff*,* desperately*,* because there’s so many other things*,* right?”* (Wave 1, criminal legal, rural).

Yet data-sharing across sectors was inconsistent. Coalitions with more diversity in community representation were more likely to leverage data successfully:

*“[HCS staff] have helped by pulling that data… some of that data comes from the sheriff’s office and some of it comes from the Board of Mental Health…making it available for everybody*,* including the coalition*,* to allow us to use that information…”* (Wave 2, criminal legal, urban*)*.

Conversely, others noted the while numerous data-related discussions took place, data did not always contribute to decisions: “*Well*,* everybody talks about how much they like data. So…I am sure*,* I know that there was…a lot of discussion about…data. I don’t know that drove too many decisions but…I know there’s a lot of discussion about data*.” (Wave 1, behavioral health, urban).

Coalitions also increasingly relied on data for advocacy and funding efforts, with confidence in using data to track community progress in implementing strategies increasing from 78.9% in 2019–2020 to 92.8% in 2023 for Wave 2. Members described data as essential for securing funding, with one participant stating,

“*I know when we were talking about funding of the of some of our projects as an agency… data was— how many people you’re going to serve*,* how many folks*,* you know what you know*,* that was a very important piece of that. I do think we drive off of data*” (Wave 1, behavioral health, urban).

However, misalignment between external funding priorities and local needs remained a challenge. Some coalition members reflected on how data can be selectively framed to meet funder expectations rather than reflect the full reality of community outcomes. As one participant explained,

“*Data tells an individual story; data is used to get grant money so there’s mixed numbers [some positive and some negative] of ‘we’re successful because we did this.’ Data can be so manipulated… I think data is often used to tell a good story instead of the real story*….” (Wave 1, criminal legal, urban).

This participant elaborated with an example from a local project where success was framed narrowly around recovery outcomes, while less favorable data—such as where participants dropped off along the care continuum—were overlooked. This reflection underscores how performance metrics may be shaped to align with external narratives, even when they obscure important aspects of community need and system gaps.

## Inner Setting: Technology Infrastructure and Learning Centeredness

All coalitions had access to the HCS dashboards and a data portal which was continually modified during the study (Fareed et al., 2024). Access to study data in Wave 1 was delayed while the tools were built while Wave 2 coalitions had immediate access. Data coordinators were available to coalitions to offer support to develop Data Action Plans. Interview findings and data coordinator reflections indicated that coalitions’ ability to use these tools effectively was shaped by their information technology infrastructure and learning-centeredness. Coalitions that actively engaged with these structured systems, including the HCS-provided community dashboard and data action planning, reported greater confidence in tracking progress and applying data to guide decisions. Those with both strong technological capacity and a supportive learning culture were more likely to implement systematic data use practices. In contrast, coalitions with limited internal capacity or less developed learning environments faced challenges in translating data into actionable strategies.

Confidence in tracking OEND implementation remained relatively stable in Wave 1 (83.3% at baseline and 80.0% at follow-up, while rose from 76.9% at baseline to 91.3% at follow-up in Wave 2. However, not all coalitions had the infrastructure or capacity to make full use of available tools, leading to underutilization and confusion in some settings.

While some coalitions had structured systems in place prior to the study, others began using data more efficiently through HCS support, particularly in developing Data Action Plans. In one coalition, this process marked a turning point in how data were understood and applied.

“*Data Action Plan and putting that together*,* in addition to the portal really helped us to kind of better understand and prioritize like data that’s essential versus data that’s just there. You know*,* and like I think that that is something that too like it was kind of a learning experience for us as well… just because*,* like knowing that all of this data is out there and knowing that you could potentially have access to it… you know*,* it helps you to kind of learn like even though it’s there it doesn’t mean that you’re actually ever going to use it*,* need it*,* or you know deal with it*,* in any kind of respect*.” (Wave 1, healthcare, urban).

Additionally, access to knowledge and information further supported coalition learning. Coalitions that leveraged structured training and regular data updates from HCS staff reported strong engagement with data. For example, when asked about the strength of partnering with HCS, one participant answered: “*Building a stream of information that is more available to practitioners and agencies*,* and you know peer supporters*,* providing education around data and how to use it.”* (Wave 1, behavioral health, urban).

In coalitions where a learning-centered culture and appropriate infrastructure coexisted, data systems were adapted based on local feedback and usability testing. One Wave 1 rural county data coordinator noted that collaborative communication and iterative revision, including trial and error with platforms such as REDCap and Smartsheet, ultimately led the community to adopt Microsoft Forms for data collection. This decision was based on considerations of timeliness, accessibility, usability, and sustainability. Despite early barriers to implementing the ideal model, the data coordinator emphasized ongoing efforts to strengthen community data use for decision-making, which contributed to more thoughtful processes and outcomes aligned with the community’s goals.

### Individuals: Capability, Confidence, and Roles in Data Engagement

Coalitions increasingly supported data-driven decision-making, yet capability shaped how individuals engaged and applied data insights. While 92.8% of members supported learning more about OEND by 2023 in Wave 2, some individuals felt as if they lacked the technical skills to interpret data effectively, but were able to receive support from more capable colleagues on the coalition:

“*I’m not real good with statistics and stuff like that*,* because I keep most of my statistics and data in my heart*,* it’s how I deal with people but*,* but I know that’s what drove us you know the needs and the data we got that’s how we knew which way to go… I don’t know statistics. [The data coordinator] knows all of that kind of stuff*,* but I feel like it’s getting better*.” (Wave 1, behavioral health, rural).

This reflection illustrates how peer support from more data-literate coalition members, particularly data champions, enabled broader participation in data-informed decision-making, even among those with limited technical expertise.

Some coalition members expressed limited confidence or comfort with data tasks, describing themselves as less inclined toward data work: *“I’m not a huge data person. I don’t know if you can tell. It’s not my forte*,* it is not my niche at all.”* (Wave 2, healthcare, urban). While this does not necessarily reflect a lack of access to support, it highlights variation in perceived capability that may influence engagement.

Even though data were made available, engagement and participation varied, suggesting challenges in motivation and opportunity among some coalition members. One Wave 2 urban county data coordinator reflected that although data were presented to the core group, comprised of key coalition decision-makers, these meetings were often poorly attended, and the data rarely prompted meaningful discussion.

When coalition members were responsible for data compilation and reporting, some important data aspects were often omitted in presentations. One data coordinator in a Wave 2 urban county reflected that coalition leadership was resistant to discussing data in depth, specifically equity-focused demographic categories for sex, race and ethnicity, and age. Further, the leadership showed reluctance toward sharing or discussing differences between groups and their outcomes in the planning of HCS efforts, even as the coalition had a stated interest in health equity and previously engaged a consultant focused on health equity. This reluctance posed a barrier to data-driven decision-making and hindered efforts to identify and address potential unmet needs and equity-related tailored interventions. In communities where coalitions considered demographic data, interventions were developed to better reach underserved demographic groups.

### Process: Reflecting on Data, Adapting Strategies, and Engaging to Support Implementation Progress

During the HCS, coalitions grew more confident in their ability to monitor opioid-related interventions, with the percentage of members who agreed or strongly agreed that their coalition is effective in tracking progress on opioid overdose reduction continuum of care approach (Opioid-overdose Reduction Continuum of Care Approach ) evidence-based practices (Winhusen et al. 2020b). Some coalitions actively used data to adapt implementation strategies, with one member stating,

“*That’s why I think [the county] has done so well because we keep track of that stuff [data] and*,* and we are flexible to… okay*,* well*,* maybe this isn’t working so well*,* well let’s switch gears and do this*.” (Wave 1, behavioral health, rural).

Having a community developed and controlled system in place to review data, such as a coalition developed website with a locally focused data dashboard, created opportunities to initiate meaningful conversations about data use. One Wave 2 rural county data coordinator reflected that the community’s dashboard development was informed by coalition data requests, and that this dashboard served as a catalyst for broader discussions within the coalition and in smaller groups. These conversations focused on identifying community-wide trends and examining how state or national events may have influenced local outcomes.

Coalition alignment with evidence-based decision-making also impacted data utilization. Some coalitions fully integrated data into their processes, stating,

*“It’s [data] guided a lot of success with that we’ve had*,* with what we’ve been doing*,* because we’re using it to make informed decisions instead of just stabbing in the dark*,* for you know*,* hoping this works or that works.”* (Wave 1, healthcare, rural).

Despite efforts to make data available and encourage its use, some coalitions noted gaps between data availability and practical use, suggesting that while coalitions were encouraged to leverage HCS and community data, some lacked the follow-through or capacity to apply it effectively. As a result, data did not consistently inform strategy evaluation or adaptation.

“*I don’t know that data drove a lot of the decisions that HCS made in our community at the end of the day… I know that there was a really big push to say like we have this you can use it*,* but I don’t know that our coalition necessarily jumped on it and started to use it*.” (Wave 1, healthcare, urban).

These findings underscore a persistent gap between data availability and actual use, highlighting the need for greater investment in building the capacity, structures, and culture required to translate data into action.

## Discussion

In this study, we applied CFIR 2.0 (Damschroder et al., 2022) to examine how multilevel contextual factors shaped community coalitions’ engagement with data during implementation of the HCS in Ohio. Using a mixed-methods design, we explored how coalitions navigated relational, organizational, and systemic influences to support data-informed decision-making related to OEND and MOUD. Our findings affirm CFIR 2.0’s emphasis on implementation as a relational and iterative process shaped by individual engagement, organizational learning, and structural alignment (Chambers et al., 2013).

Across 18 coalitions, confidence and knowledge about opioid-related data increased over time, suggesting the intervention enhanced awareness of EBPs and data literacy. Yet, greater knowledge did not always translate into strategic use. While all communities had access to data coordinators and were supported in developing a Data Action Plan, coalitions that consistently engaged with these resources and integrated data into planning with the guidance of data coordinators demonstrated more effective use than those that did not fully leverage them. For example, some identified local disparities in MOUD access using dashboard data and successfully advocated for system changes. These coalitions aligned across CFIR domains of adaptability, readiness, and integration (Glasgow et al., 2019), echoing literature on the need for user-centered data systems in community settings (Fareed et al., 2024; Olvera et al., 2024; Ryan et al., 2014). In contrast, others struggled to operationalize data use despite tool access. Barriers included limited technical capacity, inconsistent meeting structures, and a perceived disconnect between tools and daily coalition activities. This reflects prior findings that data tools alone are insufficient without supportive organizational processes and infrastructure (Fareed et al., 2024; Olvera et al., 2024; Ryan et al., 2014).

Inner setting factors such as leadership engagement, meeting structure, and a culture of learning proved important (Shelton et al., 2018). Coalitions with regular meeting schedules and standing data reviews were more likely to report ongoing engagement and adaptation based on data. These findings echo prior research showing that organizational learning climate and feedback loops enhance implementation quality (Cashman et al., 2008). In contrast, coalitions without these structures often missed opportunities to leverage data for real-time decision-making.

Outer setting influences, including data-sharing partnerships, system fragmentation, and funding alignment, played a significant role in shaping coalition behavior. Coalitions that included partners, such as law enforcement or behavioral health agencies, were better positioned to access and interpret local data. However, others cited inconsistencies in data access and tensions between funder-driven metrics and local priorities. Participants noted that data were at times framed to highlight successes, which could inadvertently obscure deeper structural challenges or equity gaps (Brownson et al., 2018), highlighting the discrepancy between accountability and equity in public health reporting.

Implementation for Wave 1 communities also overlapped with the onset of the COVID-19 pandemic, which likely disrupted coalition functioning, meeting attendance, and cross-sector coordination (Walters et al., 2021). Although we did not directly assess pandemic-related effects, COVID-19 represents an important outer-setting influence that may have shaped engagement with data systems and routine coalition processes, particularly during early implementation. This context should be considered when interpreting differences in coalition participation and data integration across waves.

Individual characteristics, especially confidence in data interpretation,^16,17,31,32^ (Katapally, 2019) were also influential in shaping coalition members’ engagement with data. While many expressed a willingness to use data, some reported limited technical skills and relied on trusted peers or data champions for support. These intermediaries played a critical role in bridging knowledge gaps and fostering engagement, reflecting the importance of relational and facilitative roles emphasized in the Community-Engaged Data Science framework. However, not all coalitions took advantage of this support, and the intervention offered limited direct opportunities to build individual capacity or increase self-efficacy with data. Although the study provided tools, dashboards, and access to data coordinators, some members still described discomfort or uncertainty when interacting with data, suggesting that technical access alone is not sufficient. Coalitions appeared to progress further when they had embedded structures for reflection, adaptation, and collective sense-making. In some communities, dashboards became catalysts for discussion and strategic shifts; in others, discomfort with data topics such as demographic stratification reduced engagement. These differences underscore the need to attend not only to the availability of data, but also to how it is shared, who facilitates interpretation, and whether individuals feel capable and supported in its use.

Findings from the Strategic Prevention Framework–State Incentive Grant (SPF-SIG) initiative similarly emphasized that coalition effectiveness depends on structured planning processes, cross-sector engagement, and sustained data infrastructure to support prevention implementation and monitoring (Johnson et al., 2017; Orwin et al., 2014). Consistent with that literature, our findings suggest that coalition-based implementation efforts require not only access to data, but also clear governance structures, role delineation, and facilitation supports to translate information into coordinated action. These parallels reinforce the importance of investing in coalition capacity and infrastructure as foundational components of community-level implementation initiatives.

Our findings reinforce that successful implementation depends not simply on the availability of data tools, but on how well those tools are integrated into the inner setting of coalition operations. Data systems, the innovation, must be adaptable, user-centered, and aligned with community workflows and goals to support meaningful use. Coalitions were most effective when data strategies matched internal infrastructure, stakeholder capacity, and shared values, and when facilitation and training extended beyond initial implementation. Transparent, equity-focused data use was fostered by policies that moved beyond compliance metrics and by data coordinators, who helped translate data into action and build trust by applying an equity lens, defined here as the intentional use of disaggregated data to identify disparities, center underserved populations, and inform strategies that address structural barriers to care. Effective data use is inherently relational and iterative, shaped by culturally attuned and technically usable design (Chambers et al., 2013).

Drawing from the HCS dashboard evaluation and the Community-Engaged Data Science model, we emphasize the need for public health initiatives to prioritize usability, narrative alignment, facilitation, and long-term stakeholder engagement (Fareed et al., 2024; Glasgow et al., 2019; Olvera et al., 2024). To strengthen community-driven, data-informed implementation, we offer several recommendations: (1) train and support embedded data champions who can contextualize and interpret information through an equity lens; (2) co-design flexible, user-informed data systems with community stakeholders to ensure relevance and responsiveness; (3) establish predictable, standing agenda time during coalition meetings for reviewing local data, supported by user-friendly visuals and facilitation guides to help normalize engagement and encourage shared interpretation; (4) promote policy and funding structures that value transparency and account for local context and complexity; and (5) sustain cross-sector partnerships to ensure ongoing access to timely, granular data beyond the life of specific grants or initiatives.

While this study offers valuable insights into coalition-based data use, several contextual factors shaped what we were able to observe and interpret. Because coalition membership evolved throughout the study period, we were not positioned to track individual-level changes over time, and each survey captured a cross-sectional snapshot of active members at that moment. Local variation in coalition structure, resource availability, and community dynamics likely influenced both implementation processes and perceptions of data use, which may affect the broader applicability of findings. Additionally, with many community-based implementation studies, our reliance on self-reported surveys, interview data, and data coordinator reflections introduces the possibility of selection bias, particularly in how those that participated characterized their knowledge, confidence, and coalition practices. We accounted for these contextual considerations in our integrated mixed-methods analytic approach but recognize that they reflect the realities of conducting research within dynamic, real-world implementation settings.

## Conclusions

Our study underscores the critical role of contextual and organizational factors in shaping how community coalitions engage with data to support opioid overdose prevention efforts. Coalitions that successfully integrated data into decision-making processes were those with strong infrastructure, cross-sector partnerships, and engagement with data coordinators to translate data into actionable strategies. Despite the availability of data tools, sustained use often depended on local capacity, leadership support, and alignment with coalition values and workflows. Guided by CFIR 2.0, these findings point to actionable strategies, such as investing in coalition-based data literacy, simplifying tool design, and fostering a culture of learning, which can enhance the implementation and sustainability of evidence-based practices within community-led public health initiatives.

## Data Availability

The datasets generated and analyzed during the current study are not publicly available due to ethical restrictions to protect participant information but are available from the corresponding author on reasonable request.

## Abbreviations

CFIR: Consolidated Framework for Implementation Research
EBPs: Evidence-Based Practices
HCS: HEALing Communities Study
MOUD: Medications for Opioid Use Disorder
OEND: Overdose Education and Naloxone Distribution
OUD: Opioid Use Disorder
